# Pediatric Type 1 Diabetes: Is Age at Onset a Determining Factor in Advanced Hybrid Closed-Loop Insulin Therapy?

**DOI:** 10.3390/jcm12216951

**Published:** 2023-11-06

**Authors:** Alfonso Lendínez-Jurado, Juan Pedro López-Siguero, Ana Gómez-Perea, Ana B. Ariza-Jiménez, Icía Becerra-Paz, Leopoldo Tapia-Ceballos, Carmen Cruces-Ponce, José Manuel Jiménez-Hinojosa, Sonsoles Morcillo, Isabel Leiva-Gea

**Affiliations:** 1 Department of Pediatric Endocrinology, Hospital Regional Universitario de Málaga, 29011 Málaga, Spain; lopez.siguero@gmail.com (J.P.L.-S.); gomezpereana@gmail.com (A.G.-P.); iciaapaz@gmail.com (I.B.-P.); leotapiaceb@hotmail.com (L.T.-C.); carmencrucesponce@gmail.com (C.C.-P.); jmjhinojosa@hotmail.com (J.M.J.-H.); isabeleivag@gmail.com (I.L.-G.); 2 Departamento de Farmacología y Pediatría, Universidad de Málaga, Andalucía Tech, Campus de Teatinos s/n, 29071 Málaga, Spain; 3 Instituto de Investigación Biomédica de Málaga (IBIMA), 29010 Málaga, Spain; sonsoles75@gmail.com; 4 Department of Pediatric Endocrinology, Hospital Universitario Reina Sofía, 14004 Córdoba, Spain; micodemas@hotmail.com; 5 Department of Pediatrics, University of Cordoba, Av. Menéndez Pidal, 7, 14004 Córdoba, Spain; 6 Instituto Maimónides de Investigación Biomédica de Córdoba (IMIBIC), 14004 Córdoba, Spain; 7 Department of Endocrinology and Nutrition, Hospital Universitario Virgen de la Victoria, 29010 Málaga, Spain; 8 CIBER in Physiopathology of Obesity and Nutrition (CIBEROBN), Instituto de Salud Carlos III Madrid, 29010 Málaga, Spain

**Keywords:** advanced hybrid closed-loop, age at onset, MiniMed^TM^ 780G, pediatric type 1 diabetes, time in hyperglycemia, time in range

## Abstract

Background: The integration of continuous glucose monitoring systems with insulin infusion pumps has shown improved glycemic control, with improvements in hyperglycemia, hypoglycemia, Hb1Ac, and greater autonomy in daily life. These have been most studied in adults and there are currently not many articles published in the pediatric population that establish their correlation with age of debut. Methods: Prospective, single-study. A total of 28 patients (mean age 12 ± 2.43 years, 57% male, duration of diabetes 7.84 ± 2.46 years) were included and divided into two groups according to age at T1D onset (≤4 years and >4 years). Follow-up for 3 months, with glucometric variables extracted at different cut-off points after the start of the closed-loop (baseline, 1 month, 3 months). Results: Significant improvement was evidenced at 1 month and 3 months after closed-loop system implantation, with better glycemic control in the older age group at baseline at TIR (74.06% ± 6.37% vs. 80.33% ± 7.49% at 1 month, *p* < 0.003; 71.87% ± 6.58% vs. 78.75% ± 5.94% at 3 months, *p* < 0.009), TAR1 (18.25% ± 4.54% vs. 14.33% ± 5.74% at 1 month, *p* < 0.006; 19.87% ± 5.15% vs. 14.67% ± 4. 36% at 3 months, *p* < 0.009) and TAR2 (4.75% ± 2.67% vs. 2.75% ± 1.96% at 1 month, *p* = 0.0307; 5.40% ± 2.85% vs. 3% ± 2.45% at 3 months, *p* < 0.027). Conclusions: the use of automated systems such as the MiniMed^TM^780G system brings glucometric results closer to those recommended by consensus, especially in age at T1D onset >4 years. However, the management in pediatrics continues to be a challenge even after the implementation of these systems, especially in terms of hyperglycemia and glycemic variability.

## 1. Introduction

Type 1 diabetes (T1D) continues to pose an elevated risk for mortality and cardiovascular disease. Patients who develop type 1 diabetes between 0 and 10 years of age have a hazard ratio of 4.11 (95% CI 3.24–5.22) for all-cause mortality, 7.38 (3.65–14.94) for cardiovascular mortality, 3.96 (3.06–5.11) for non-cardiovascular mortality, 11.44 (7.95–16.44) for cardiovascular disease, 30.50 (19.98–46.57) for coronary heart disease, 30.95 (17.59–54.45) for acute myocardial infarction, 6.45 (4.04–10.31) for stroke, 12.90 (7.39–22.51) for heart failure and 1.17 (0.62–2.20) for atrial fibrillation [1,2,3]. Overall, the development of type 1 diabetes before the age of 10 years results in a loss of 17.70 years of life (95%CI 14.50–20.40) for women and 14.20 years of life (12.10–18.20) for men [1,4]. These data may justify an increased focus on cardioprotection in people with early-onset T1D [5,6,7].

Closed-loop systems offer the greatest benefit in terms of time in range (TIR) and protection against hypoglycemia and hyperglycemia [8,9,10,11], which may decrease cardiovascular risk and long-term mortality.

One of the closed-loop systems is the Medtronic MiniMed^TM^780G device. It features an advanced hybrid closed-loop (AHCL) algorithm that includes automatic delivery of basal insulin every 5 min, adjustable targets of 100 (5.50), 110 (6.10) and 120 (6.70) mg/dL (mmol/L), and automatic delivery of correction boluses every 5 min. Meal information must be input by the user or caregiver. Autocorrection every 5 min improves daytime blood glucose by mitigating inaccuracies in carbohydrate estimation and late or missed meal boluses, and adapts to interday glucose variability without user intervention. This system was approved by the European Commission in June 2020 and is indicated for individuals with T1D aged 7 to 80 years, whose minimum total daily insulin dose is 8 U/day [12].

Given the limited number of studies of MiniMed^TM^780G system in children and that thus far its correlation with age at debut has not been clearly established, we aimed to analyze whether age at onset influences glycemic outcomes after implantation of this AHCL system.

## 2. Materials and Methods

This was a prospective, single-center study carried out in the diabetes unit of a tertiary hospital in Spain (Hospital Regional Universitario de Malaga) and based on data retrieved from digital medical records during the 6-month follow-up period. We included pediatric and adolescent patients aged 7 to 17 years, diagnosed with T1D according to the latest ADA and ISPAD criteria [13,14], who were on combined treatment with continuous subcutaneous insulin infusion (CSII) (MiniMed^TM,^ Medtronic MiniMed, 18000 Devonshire Street, Northridge, CA 91325, USA) and intermittently scanned continuous glucose monitoring (isCGM) (FreeStyle Libre 2^®^ Abbott Diabetes Care 1360 South Loop Road Alameda, CA 94502, USA) and had this system replaced with the MiniMed^TM^780G AHCL system with Guardian 4^TM^ sensor (G4S) between December 2021 and April 2022. All the patients were followed by a multidisciplinary team comprising pediatric endocrinologists and diabetes nurses. Those with an interstitial glucose sensor system other than the one previously described were excluded.

Metabolic control variables were extracted using the LibreView^®^ (Abbott Diabetes Care 1360 South Loop Road Alameda, CA 94502, USA) and CareLink^®^ (Medtronic MiniMed, 18000 Devonshire Street, Northridge, CA 91325, USA) download platforms at the start of the study and at different cut-off points after reaching a daily time greater than 90% in auto mode (1 month and 3 months). Baseline sensor and insulin pump data were downloaded for the 2 weeks prior to the implementation of the AHCL using the Libreview and Carelink System platforms, respectively.

The variables studied were as follows:

- Time in Range (TIR): percentage of time in which interstitial blood glucose levels are between 70 and 180 mg/dL.
- Time Above Range 1 (TAR1): percentage of time in which interstitial blood glucose levels are between 180 and 250 mg/dL.
- Time Above Range 2 (TAR2): percentage of time in which interstitial blood glucose is above 250 mg/dL.
- Time Below Range 1 (TBR1): percentage of time in which interstitial blood glucose levels are between 70 and 54 mg/dL.
- Time Below Range 2 (TBR2): percentage of time in which interstitial blood glucose is below 54 mg/dL.

Other parameters included were the percentage of time in auto mode, coefficient of variation (CV), daily insulin dose (U/kg/day), percentage of insulin in basal or bolus form, the amount of insulin administered as automatic correction, glycated hemoglobin (Hb1Ac), glucose management indicator (GMI) and mean glucose.

Quality of life variables (sleep and satisfaction with treatment) were also studied through questionnaires completed in person by the main caregivers both at baseline and 3 months after initiation of the closed-loop system.

- Pittsburgh Sleep Quality Index (PSQI): The sum of the scores of the 19 questions, or the total score, indicates the overall sleep quality of the person being evaluated. This total score can range from 0 to 21 points. The higher the total score, the worse the sleep quality. Thus, a total score less than or equal to five on the Pittsburgh scale indicates that, in general, sleep quality is optimal, while a total score greater than five suggests sleep disturbances, of greater or lesser severity [15].
- Diabetes Treatment Satisfaction Questionnaire (DTSQ) index: assesses satisfaction (status version, DTSQ-s) and change in satisfaction (change version, DTSQ-c) with treatment for T1D. DTSQ-s scores range from 0 to 48, and DTSQ-c scores range from −24 to +24, with higher scores indicating greater satisfaction [16].

To test the relationship between age at onset and the above variables, patients were divided into two groups: ≤4 years (group 1), >4 years (group 2) [17].

The study protocol was in accordance with the Declaration of Helsinki. All participants and their caregivers were informed of the study and signed a consent form. The included patient under 7 years was 6 years and 6 months old. The patient’s parents were informed of the gap of 6 months from the minimum age limit for device use. The protocol was approved by the ethics committee of our center.

Data analysis was performed using free R 4.0.2 software (R-CoreTeam 2020) (https://www.r-projetc.org/, accessed on 20 October 2022). A Shapiro–Wilk test analysis was performed to determine the normality of the study variables. Results are presented as mean ± SD values in normal distributions or as median (IQR) in non-normal distributions. A Wilcoxon signed-rank test was performed to analyze differences in the non-normal distributions, and the paired *t*-test was used in the normal distributions. Correlation analyses were performed using Pearson’s correlation coefficient in parametric variables and Spearman’s correlation coefficient in non-parametric variables. A chi-square test was used to perform a bivariate analysis. A *p*-value < 0.05 was considered statistically significant. *p*-values were adjusted using the Benjamini–Hochberg correction for multiple comparisons.

## 3. Results

All figures for each of the glycemic outcomes and system usability variables and their *p*-value are shown in Table 1.

### 3.1. Study Population Characteristics

A total of 28 patients were included, 57.14% of whom (16 patients) belonged to group 1 (age at onset ≤4 years), and the remaining 42.86% (12 patients) belonged to group 2 (>4 years). The mean age at diabetes debut in group 1 was 2.46 ± 1.05 years and 6.43 ± 1.68 years in group 2. The median age at MiniMed^TM^780G device initiation was 11.15 ± 2.73 years in group 1 and 13.14 ± 1.38 years in group 2; median time from debut to initiation of the closed-loop system was 8.69 ± 2.75 years and 6.71 ± 1.45 years, respectively.

### 3.2. System Usability

The median total insulin before the system change was 0.87 U/kg/day (group 1) and 0.81 U/kg/day (group 2). We observed a non-significant increase in the total amount of insulin in both groups during follow-up. No differences were observed either at 1 month (*p* = 0.4344) or at 3 months (*p* = 0.6474) when comparing the percentage of autocorrection in the two groups.

We calculated the mean glucose obtained with CSII-isCGM and after initiation of the new system. Significant differences were only observed between the two age groups at baseline (*p* < 0.003).

Baseline HbA1c was 7.34% in group 1 and 6.68% in group 2, and at 3 months 6.94% and 6.65%, respectively. When assessing the impact of age at onset on this parameter, we observed significant differences between the two groups at baseline (*p* < 0.011), although this was not so 3 months after closed- loop implantation (*p* = 0.5082).

Regarding CV, we found that, at baseline, patients had a percentage of up to 39.83% (group 1) and 38.03% (group 2). Analyzing the relationship with age at onset, only differences in CV were observed between the two groups at 3 months (*p* < 0.05).

### 3.3. Glycemic Outcomes

**Figure 1:**
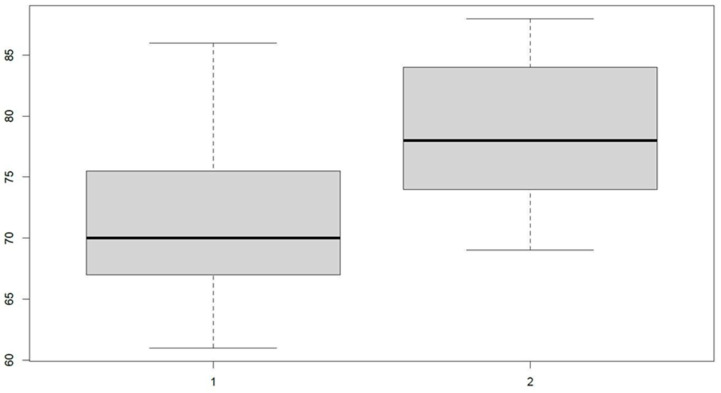
Percentage of TIR at 3 months (Y-axis) after closed-loop implantation, categorizing both groups according to age at diabetes onset (X-axis) (1. ≤4 years; 2. >4 years).

When assessing TIR, we observed that after the implementation of the closed-loop system, there was a significant increase in the average percentage of this value during the follow-up, in both age groups. When evaluating the relationship between age at onset and TIR, significant differences were observed between the two groups at all cut-off points (baseline *p* < 0.0004; 1 month *p* < 0.03 (Figure 1); 3 months *p* < 0.009).

A statistically significant and directly proportional relationship was observed between age at onset and TIR at 3 months (R^2^ = 0.1784, *p* < 0.0282), which was not significant when comparing TIR with duration of diabetes (*p* = 0.431) and age at AHCL device installation (*p* = 0.147).

With respect to TAR1, we noted a decrease after the implementation of the new system, both at one and three months, and it was also statistically different depending on the age of onset (1 month *p* < 0.006; 3 months *p* < 0.009).

Concerning TAR2, our patients presented a reduction of this percentage both after one month and three months of the new system. Significant differences were observed at all cut-off points when evaluated according to age groups at debut (1 month *p* = 0.0307; 3 months *p* < 0.027).

In relation to TBR1, no significant differences were observed at any of the cut-off points studied (baseline *p* = 0.6041; 1 month *p* = 0.7915; 3 months *p* = 0.6314).

Finally, when TBR2 was assessed, no significant differences were observed at any of the cut-off points studied (baseline *p* = 0.8913; 1 month *p* = 0.6216; 3 months *p* = 0.948).

### 3.4. Quality of Life

The mean score for the DTSQ prior to the change in system (DTSQ-s) was 37.62 (IQR 37–39) in group 1 and 33.91 (IQR 33-35) in group 2. Three months after its implementation (DTSQ-c), a mean score of 13.50 (11.75–15) was obtained in group 1 and 12.25 (IQR 9–15.75) in group 2.

In relation to the DTSQ prior to the system change (DTSQ-s), the mean score obtained was 37.62 (IQR 37–39) in group 1 and 33.91 (IQR 33–35) in group 2. At 3 months after closed-loop system implantation (DTSQ-c), a score of 13.50 (11.75–15) was obtained in group 1 and 12.25 (IQR 9-15.75) in group 2. When studying the impact of age at onset on treatment satisfaction, significant differences were observed with the CSII-isCGM treatment (*p* < 0.016), but not with the MiniMed^TM^780G system (*p* = 0.5954).

The questionnaire on the sleep quality of both parents (PSQI) showed a baseline value of 4.91 (IQR 2–6.50) in group 1 and 8.44 (IQR 4–12) in group 2. Three months after the implementation of the new system, the result for group 1 was 4.14 (IQR 3.50–4.05) and, for group 2, it was 5.75 (IQR 2–10). No statistically significant improvements were observed either at baseline (*p* = 0.1886) or at 3 months (*p* = 0.8577).

## 4. Discussion

This study is the first to correlate age at T1D onset with outcomes after AHCL implantation.

Our study shows more favorable results in the achievement of consensus targets in patients with T1D with onset after 4 years of age, both at 1 and 3 months after AHCL system implantation. At baseline before closed-loop system implantation, significant differences in mean glucose percentage, TIR, TAR1 and TAR2 were found between these two groups. After implantation of the MiniMed^TM^780G system, there was an increase in TIR in both groups, with both groups achieving consensus targets (>70%). Nevertheless, significant differences remained between the two groups at the different cut-off points (1 month and 3 months) in favor of the patients who were over 4 years of age at onset.

Regarding TAR1 there were also significant differences between the groups at baseline. The implementation of AHCL enabled the mean consensus-recommended percentage of TAR1 to be met in both groups, although with significant differences between the two groups, with a lower TAR1 in the patients who were over 4 years of age at onset, both 1 and 3 months of follow-up.

In TAR2 there were also significant differences at baseline, with neither group reaching the consensus target. The use of the Medtronic system allowed the consensus-recommended percentage of TAR2 (<5%) at 1 month to be met in both groups, although with significant differences in favor of better control in the group over 4 years of age at onset. These significant differences were maintained at 3 months. In addition, at this cut-off point, the group with onset before the age of 4 years had a higher mean TAR2 than the consensus recommendation.

There were no differences between these two groups at baseline or at follow-up in hypoglycemia parameters (TBR1 and TBR2). There were significant differences in glycemic variability measured by CV at 3 months after AHCL implantation, with greater variability in the group with onset before 4 years of age, although the figures are very close to those recommended.

Published studies on children with AHCL systems do not classify by age at onset as established in our work, but by age at implantation. One example is the study by Ng et al., in which a group of 251 children with a median age of 12.30 years (IQR 2-19) using AHCL systems (Tandem t:slim X2, Medtronic MiniMed™780G and CamAPS FX) showed improvements in glycemic control, TIR, percentage of hypoglycemia, fear of hypoglycemia and sleep quality when using AHCL therapy for 6 months. Fear of hypoglycemia and sleep quality also improved for their parents and caregivers at 6 months. These data are consistent with our results of increased TIR and improved sleep quality [11].

In the study by Arrieta et al., results at the first month of follow-up in users aged 15 years or younger (*n* = 3211) were an estimated HbA1c 6.80% ± 0.30% and TIR 73.90% ± 8.70. The TBR was within the consensus target of less than 4% (3.20%). These results were maintained during the 6-month follow-up [18]. The first group we found that classified groups by age at AHCL implantation is that of Karakuş et al., which collected data from 4193 pump days and continuous glucose monitoring of 34 children using the MiniMed^TM^780G system. Microboluses and autocorrection boluses were analyzed hourly for two age groups: under 9 years and over 9 years. The proportion of microboluses was significantly higher in children younger than 9 years than in children older than 9 years (*p* < 0.003) [7].

Tornese et al. retrospectively evaluated 44 T1D pediatric users (2–21 years) with the MiniMed™670G system (SHCL) and the 780G system (AHCL) at baseline and after 3 and 6 months. They concluded that children under 14 years of age appear to benefit the most from the AHCL systems, as do people with the worst glycemic control. Significant differences in HbA1c were found between SHCL and AHCL users in children aged 7–14 years (7.70% vs. 7.10% at 6 months) and in those with worse glycemic control (HbA1c > 8%) at baseline (8.10% vs. 7.10% after 6 months). All sensor-specific measures of glycemic control improved from manual to automatic mode in both SHCL and AHCL users, with no increase in time to hypoglycemia. However, the percentage of patients with TIR > 70% increased significantly in AHCL users [19,20]. In our study, we demonstrate an earlier achievement of the TIR target after implantation (1 month) and add that children older than 4 years at debut achieve a greater improvement in TIR, TAR1, TAR2 and CV than those younger than 4 years.

Piccini et al. showed that the AHCL MiniMed™780G allowed rapid and sustained improvement of glycemic control in 44 children with T1D. Adolescents had good adherence to this technology with optimal TIR, which was better maintained over time compared to younger children. Tighter glycemic target settings were associated with better metabolic control, with no increase in severe hypoglycemia episodes. The mean TIR at 14 days in automatic mode was 76.30% ± 9.60% vs. 69.30% ± 12.60% in manual mode (*p* < 0.001), and this improvement was maintained for 6 months. HbA1c was 7.20% ± 0.70% (55 mmol/mol) at baseline and improved significantly after 3 months (6.70% ± 0.50%, 50 mmol/mol, *p* < 0.001) and 6 months (6.60% ± 0.50%, 49 mmol/mol, *p* < 0.001). TIR was higher in individuals >13 years at all time periods (*p* < 0.001). A glycemic target <120 mg/dL was associated with better TIR [21].

In a literature review of the last 10 years, we found that most of the studies focused on the age at device implantation without taking into account age at debut. Some studies are beginning to mention differences in metabolic control of T1D according to age at onset [17]. This theory could be explained by differences in C-peptide leakage. The percentage of individuals with stimulated C-peptide ≥0.20 nmol/L or detectable C-peptide ≥0.017 nmol/L decreases during the first 4 years after debut, and is markedly influenced by age of disease onset with negative results observed in the pediatric population. According to the study by Hao et al., only 5% maintained their baseline C-peptide secretion at 4 years. The expected inverse relationships between C-peptide and HbA1c or insulin doses varied with time course and age at onset. Combined clinical variables, such as insulin dose-adjusted HbA1c and its ratio to C-peptide, were also influenced by age at presentation and time since diagnosis [22]. Other studies have age at onset cut-off points below 7 years, when compared to groups aged 7 to 12 or more than 12 years, with significant differences in the loss of insulin-positive beta cell-containing islets within 5 years of diagnosis [23].

In recent years, we have observed epidemiological changes, with a statistically significant increase in the presentation of T1D in Spain in the youngest age group, represented by ages under 4 years, with respect to previous periods 2015–2019 [17]. The epidemiological change coupled with a significant increase in the under-4 age group has led us to focus on the metabolic control of this age group with the most advanced systems. This group of patients with very early onset is an emerging group that represents an important therapeutic challenge due to the increased cardiovascular risk. Metabolic memory plays an important role due to the potentially longer duration of the disease and the early loss of C-peptide, which could lead to greater difficulty in metabolic control [24].

## 5. Conclusions

This study enables us to focus on prioritizing the group of patients who debut at an earlier age, as they represent a challenge in metabolic control. Undoubtedly, the use of automated systems such as the MiniMed^TM^780G system brings glucometric results closer to those recommended by consensus. However, the management of this group of patients continues to be a challenge even after the implementation of these systems, especially in terms of hyperglycemia and glycemic variability. Further development and analysis of automated algorithms in younger patients is necessary. These patients have a strong need for glycemic accuracy to combat metabolic memory and an increased cardiovascular risk that is closely linked to earlier age at debut.

## Figures and Tables

**Table 1:** Glycemic outcomes and system usability categorized by age groups after 3 months of use of an advanced hybrid closed-loop system compared to baseline (continuous subcutaneous insulin infusion and flash glucose monitoring).

	Baseline (CSII + isCGM)		*p*-Val	1 Month (AHCL—Automatic Mode)		*p*-Val	3 Months (AHCL—Automatic Mode)		*p*-Val
	Group 1 (≤4 year)	Group 2 (>4 year)		Group 1 (≤4 year)	Group 2 (>4 year)		Group 1 (≤4 year)	Group 2 (>4 year)	
TIR (%) Mean ± SD	53.07 ± 9.44	67.42 ± 8.71	*p* < 0.0004	74.06 ± 6.37	80.33 ± 7.49	*p* < 0.03	71.87 ± 6.58	78.75 ± 5.94	*p* < 0.009
TAR1 (%) Mean ± SD	27 ± 6.21	21.25 ± 4.63	*p* < 0.011	18.25 ± 4.54	14.33 ± 5.74	*p* < 0.006	19.87 ± 5.15	14.67 ± 4.36	*p* < 0.009
TAR2 (%) Mean ± SD	16.27 ± 9.14	6.92 ± 4.89	*p* < 0.003	4.75 ± 2.67	2.75 ± 1.96	*p* = 0.0307	5.40 ± 2.85	3 ± 2.45	*p* < 0.027
TBR1 (%) Median (IQR)	3.07 (0.50–3.50)	3.75 (2–5.25)	*p* = 0.6041	2.25 (1–3.25)	2.08 (1–3)	*p* = 0.7915	2.27 (1–3)	2.50 (2–3)	*p* = 0.6314
TBR2 (%) Median (IQR)	0.60 (0–0.50)	0.67 (0–1)	*p* = 0.8913	0.69 (0–1)	0.50 (0–1)	*p* = 0.6216	0.60 (0–1)	0.58 (0–1)	*p* = 0.948
Glucose (mg/dL) Median (IQR)	177.47 (169.50–186.50)	153 (143.75–159.50)	*p* < 0.003	144.81 (139–150)	141.92 (127.75–149.50)	*p* = 0.6569	153.67 (138.50–156.50)	139.17 (132–147.50)	*p* < 0.074
GMI (%) Mean ± SD	7.46 ± 0.47	6.99 ± 0.30	*p* < 0.036	6.80 ± 0.21	6.60 ± 0.29	*p* = 0.1709	6.87 ± 0.26	6.64 ± 0.27	*p* = 0.1
HbA1c (%) Mean ± SD	7.34 ± 0.64	6.68 ± 0.55	*p* < 0.011	-	-	-	6.94 ± 0.56	6.65 ± 0.70	*p* = 0.5082
CV glucose (%) Mean ± SD	39.83 ± 5.54	38.03 ± 4.84	*p* = 0.3741	36.61 ± 3.75	33.60 ± 4.06	*p* = 0.0574	36.72 ± 3.46	34.11 ± 3.09	*p* < 0.05
Parameters of use									
Total daily insulin (U/kg/day) Median (IQR)	0.87 (0.63–1.06)	0.81 (0.64–1.01)	*p* < 0.7414	1.04 (0.80–1.22)	0.93 (0.76–1.10)	*p* = 0.3653	1.08 (0.82–1.15)	0.95 (0.78–1.06)	*p* = 0.8470
Total basal insulin (%) Mean ± SD	38.19 ± 11.22	40.83 ± 9.97	*p* = 0.7298	39.50 ± 6.95	38.92 ± 5.38	*p* = 0.424	38.93 ± 6.46	38.25 ± 6.14	*p* = 0.8948
Total bolus insulin (%) Mean ± SD	61.81 ± 11.22	59.17 ± 9.97	*p* = 0.7298	60.50 ± 6.95	61.08 ± 5.38	*p* = 0.424	61.07 ± 6.46	61.75 ± 6.14	*p* = 0.8948
Total autocorrection insulin (%) Mean ± SD	-	-	-	24.25 ± 7.57	19.67 ± 9.60	*p* = 0.4344	25.53 ± 8.15	21.92 ± 7.75	*p* = 0.6474
Fingerstick BG/day Mean ± SD	3.81 ± 2.30	1.76 ± 1.47	*p* < 0.0451	0.70 ± 0.82	0.53 ± 0.33	*p* = 0.4396	0.71 ± 0.84	0.58 ± 0.48	*p* = 0.5116
Sensor use (%) Median (IQR)	89.27 (89.50–98.50)	88.50 (88–97.25)	*p* = 0.5593	97.19 (96–98)	96.75 (96.50–98)	*p* = 0.596	95.80 (96–98)	96.50 (95.75–97.25)	*p* = 0.8229
Time in Auto Mode (%) Median (IQR)	-	-	-	99.06 (98.75–100)	99.17 (98.75–100)	*p* = 0.8675	97.47 (98–100)	98.58 (97.75–100)	*p* = 0.4948

## Data Availability

All data are available through contacting the authors.
